# Current Vaccine Platforms in Enhancing T-Cell Response

**DOI:** 10.3390/vaccines10081367

**Published:** 2022-08-21

**Authors:** Takehiro Ura, Masaki Takeuchi, Tatsukata Kawagoe, Nobuhisa Mizuki, Kenji Okuda, Masaru Shimada

**Affiliations:** 1 Department of Ophthalmology and Visual Science, Graduate School of Medicine, Yokohama City University, Yokohama 236-0004, Japan; 2 Department of Ophthalmology and Visual Science, School of Medicine, St. Marianna University, Kawazaki 216-8511, Japan; 3 Department of Molecular Biodefense Research, Graduate School of Medicine, Yokohama City University, Yokohama 236-0004, Japan

**Keywords:** T-cell-mediated immunity, vaccines, infectious diseases, viral vectors, mRNA vaccines

## Abstract

The induction of T cell-mediated immunity is crucial in vaccine development. The most effective vaccine is likely to employ both cellular and humoral immune responses. The efficacy of a vaccine depends on T cells activated by antigen-presenting cells. T cells also play a critical role in the duration and cross-reactivity of vaccines. Moreover, pre-existing T-cell immunity is associated with a decreased severity of infectious diseases. Many technical and delivery platforms have been designed to induce T cell-mediated vaccine immunity. The immunogenicity of vaccines is enhanced by controlling the kinetics and targeted delivery. Viral vectors are attractive tools that enable the intracellular expression of foreign antigens and induce robust immunity. However, it is necessary to select an appropriate viral vector considering the existing anti-vector immunity that impairs vaccine efficacy. mRNA vaccines have the advantage of rapid and low-cost manufacturing and have been approved for clinical use as COVID-19 vaccines for the first time. mRNA modification and nanomaterial encapsulation can help address mRNA instability and translation efficacy. This review summarizes the T cell responses of vaccines against various infectious diseases based on vaccine technologies and delivery platforms and discusses the future directions of these cutting-edge platforms.

## 1. Introduction

A higher safety profile and induction of T-cell-mediated immunity are current trends in vaccine development. Elucidating the biological immune response enables clarification of the mechanism of action of the vaccine. The most effective vaccine immunity is likely to induce both cellular and humoral immune responses against pathogens. The assessment of T-cell responses has recently become popular in the early phases of clinical trials. The immune properties of T cells determine the efficacy of vaccines associated with durable immunity and the induction of broadly neutralizing antibodies. For example, an increasing number of follicular helper T cells (Tfh) confer long-term antibody production, which correlates with vaccine efficacy [1,2].

Traditional vaccines have been developed as live-attenuated formulations with weakened pathogens. Since then, injectable inactivated or subunit vaccines have been developed as more stable formulations with no risk of infection [3]. Conjugate vaccines with bacterial polysaccharides bound to carrier proteins have been widely used as antibacterial vaccines [4]. Most inactivated vaccines are designed with a focus on producing neutralizing antibodies to prevent infection. For example, the criteria for influenza vaccine are based on hemagglutination-inhibition antibody titers in the serum. The frequencies of HA-specific CD4 T cells are correlated with the production of HA-specific antibodies [5]. However, the 2009 H1N1 influenza pandemic gave us the opportunity to reconsider another role of T-cell-mediated immunity. The prevalence of pre-existing influenza antigen-specific T cells has been found to be associated with less severe illness [6,7]. This observation indicates that providing T-cell-mediated vaccine immunity may contribute to reducing the severity of infections. Moreover, cross-reactive T cell-mediated immunity is necessary to reduce the risk of antibody-dependent enhancement (ADE). In the development of respiratory syncytial virus (RSV) and dengue virus (DENV) vaccines, the production of non-neutralizing antibodies exacerbates viral infection, leading to enhanced disease after subsequent infection [8].

Viral vectors and mRNA vaccines are technological platforms that can induce both antigen-specific humoral and cellular immunity [3]. These platforms allow for the delivery of the antigen of interest to cells and their subsequent uptake into antigen-presenting cells (APCs). Due to their rapid and low-cost manufacturing, these vaccines are currently used in the ongoing corona virus disease 2019 (COVID-19) pandemic. Viral vectors can induce robust cytotoxic T lymphocytes (CTLs) and have been considered suitable for vaccines against retroviral infections such as the human immunodeficiency virus type 1 (HIV-1) [9]. In the development of mRNA vaccines, improvements in mRNA stability and intracellular delivery have been required. To address the technical problems associated with these vaccines, mRNA modifications and lipid nanoparticles (LNPs) have been developed. Antigen encapsulation technology with novel nanomaterials has also been applied to recombinant protein- or peptide-based vaccines [10].

Weak immunogenicity is sometimes a problem with inactivated vaccines. The combined use of adjuvants and biomaterials helps to enhance the immunogenicity of vaccines. The administration route also influences immunogenicity. Tissue-targeted delivery enhances immunogenicity and induces local immunity. Tissue-resident memory T-cell (Trm) induction is required to respond rapidly upon re-exposure to antigens [11]. Therefore, optimized administration routes with novel medical devices are needed for the spread of vaccines [12,13]. The activation and regulation of T cells determines the efficacy of the vaccine as multiple functions have been reported for the role of T cells. In this review, we elaborate on the T-cell responses of vaccines against various infectious diseases based on vaccine technologies and delivery platforms. We also discuss the future directions of these cutting-edge platforms.

## 2. T-Cell Function and Regulation

**Figure 1:**
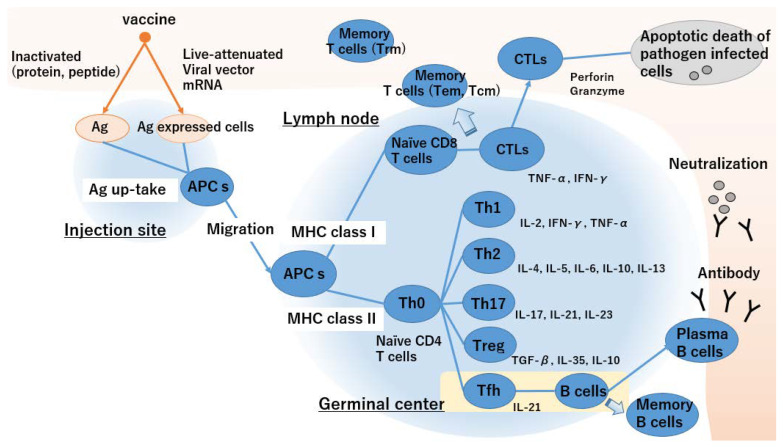
Generation of humoral and cellular immunity by vaccines.

Figure 1 illustrates the generation of cellular and humoral immunity by vaccines. Vaccine immunology is based on an adaptive immune response mediated by T cells (cellular immunity) and B cells that produce antibodies (humoral immunity).

1. APC takes up an inactivated vaccine and loaded MHC class II as extrinsic antigens and antigen-expressing cells and loaded MHC class I as endogenous antigens.
2. Activated APCs migrate towards the lymph nodes and present antigens to T cells via MHC class II.
3. Antigen presentation via MHC class II activates naïve CD4^+^ T cells and promotes Th2 cell differentiation. In contrast, antigen presentation via MHC class I activates naïve CD4^+^ and CD8^+^ T cells and biased Th1 cell differentiation.
4. Differentiation of naïve T cells depends on the cytokine environment of the microenvironment.
5. Tfh cells enhance the humoral immune response and B cell antibody production. Plasma B cells circulate throughout the body, and neutralizing antibodies prevent infection.
6. Antigen specific CTLs circulate throughout the body and kill pathogen-infected cells.
7. Memory B and T cells maintain immunosurveillance and confer long-term protective vaccine immunity.

The generation of adaptive immune responses is important for the control and clearance of pathogenic infections. The T-cell response determines the efficacy of vaccines associated with durable immunity and the induction of broadly neutralizing antibodies. T cells are categorized as either cytotoxic CD8^+^ T cells (also called CTLs) or helper CD4^+^ T cells (Th cells). Th cells are categorized into subsets based on their cytokine production profiles. Distinct Th-cell differentiation is programed depending on cytokines in the microenvironment.

### 2.1. Cellular Immunity

Cellular immunity, involving cells such as CTLs, recognizes CD8^+^ T-cell antigen epitopes and plays a pathological role during infection. The existence of pathogen-specific cellular immunity plays a critical role in eliminating the intracellular parasitic infection of bacteria. It is also essential for the control of viral infections, including influenza virus, poxviruses, coronavirus, and herpes viruses [14]. In the case of human papilloma virus (HPV) infection, the expression of oncogenic viral proteins is involved in the malignant transformation of HPV-associated tumors. Thus, the induction or transformation of HPV-specific CTLs has potential in therapeutic anti-tumor vaccines [15]. In HIV-1 infection, antiviral CTLs correlate with the clearance of virus particles that are associated with disease progression [16]. In the case of retrovirus infection, eliminating infected cells is crucial because the retrovirus integrates its genome into the host cell DNA and has a high mutation rate [16]. The mechanism of inducing CTLs follows, while APCs take up pathogen-infected cells. The pathogens are degraded to pathogen-derived peptides and loaded onto the surface of major histocompatibility complex (MHC) class I molecules as endogenous antigens. Consequently, naïve CD8^+^ T cells differentiate into CTLs through T-cell receptor (TCR) recognition of MHC class I-loaded peptides on APCs. CTLs produce perforin, granzyme, and antiviral cytokines such as interferon-gamma (IFN-γ) and tumor necrosis factor-α(TNF-α), inducing apoptotic death in pathogen-infected cells [17].

### 2.2. Humoral Immunity

Antibody-mediated humoral immunity protects against extracellular pathogens. It is well known that passive transport of maternal antibodies across the placenta protects the newborn against a wide variety of pathogens [18]. The humoral immune response magnitude is associated with disease severity [19]. The mechanism of humoral immunity induction follows. When a pathogen invades the tissue, APCs take up and recognize the pathogen as an exogenous antigen. Activated APCs migrate to the draining lymph node, where they present the peptide antigen to T cells through MHC class II on their surface. MHC class II antigen presentation activates CD4^+^ T cells and augments cytokine secretion. In specific CD4^+^ T-cell subsets, Tfh cells help B cell proliferation and maturation of the humoral response to increase antibody affinity for the antigen.

### 2.3. Th Cells

Once Th cells recognize MHC-loaded peptide antigens on APCs, they begin to divide into various effector cells. Th1, Th2, and Th17 are three major CD4^+^ T cell subsets that augment and coordinate the functions of antipathogenic immunity.

Th1 cells, which produce large quantities of IFN-γ and interleukin (IL)-12, are involved in the clearance of certain intracellular pathogens. These Th1 cytokines promote the activation of monocytes and macrophages as well as the development of CTLs, which are responsible for cellular immunity. Th1-dominant antiviral immunity plays a role in moderating the severity of infectious diseases [20].

Th2 cells produce various ILs such as IL-4, -5, -6, -10, and -13 that regulate eosinophils, basophils, and mast cells. IL-4 is a Th2 cytokine that augments the humoral immune response and antibody production by B cells. It is now clear that Tfh cells, not Th2 cells, play a critical role in the formation and function of B cell responses. IL-21 producing Tfh cells are located in the germinal center (GC) of secondary lymphoid organs. GC Tfh cells also produce IL-4, which is required for optimal B cell activation [1,2]. Hence, the induction of Tfh cells is associated with vaccine immunogenicity and humoral immunity.

Th17 cells producing IL-17A, -17F, and -22 play important roles in the clearance of cellular pathogens at the mucosal site [21,22]. Th17 cytokines involved in the regulation of neutrophils and IL-17 control the production of IFN-γ, which is necessary for the enhancement of the Th1 response. Th17 cells are necessary for the maintenance of mucosal immunity, which controls microbial translocation. Th17 cells contribute to the control of chronic HIV-1 infection and the progression to AIDS.

Regulatory T cells (Tregs) are a unique T-cell subset that plays a major role in immune tolerance. They secrete large amounts of IL-10, which limits T-cell activation and differentiation. The enhancement of antigen-specific Tregs impairs pathogen control and interferes with vaccine immunity [23].

In vaccination, formulations, adjuvants, and delivery methods affect the polarization of the Th response and determine its efficacy. Currently, many technical platforms for vaccines have been developed to induce T-cell-mediated immunity.

### 2.4. Memory Cells

Over the weeks after vaccination, the majority of activated T and B cells die, and a portion of the activated cells differentiate into memory cells. The presence of immunological memory cells also contributes to the moderate severity of infectious diseases. For example, the presence of cross-reactive pre-existing CD4^+^ T cells in a population of older adults contributed to the prevention of severe influenza symptoms during the 2009 H1N1 pandemic [6,7]. Pre-existing cross-reactive T-cell-mediated immunity enhances immune responses against severe acute respiratory syndrome coronavirus 2 (SARS-CoV-2) infection and moderates the severity of COVID-19 [24,25,26,27]. In the case of COVID-19, 40–60% of unexposed individuals possess virus-specific T-cell immunity due to prior exposure to common coronaviruses, which may contribute to less severe disease progression in some populations of patients with COVID-19 [28,29].

Memory T cells are divided into several subpopulations. Effector memory T cells (Tem) continually recirculate between tissues and blood via the lymph to maintain immunosurveillance [30]. Central memory T cells (Tcm) surveil secondary lymphoid organs. Recently, memory T cells that reside in the frontline sites of an infection, such as the skin, lung, and gastrointestinal tract, without recirculation, have been found. These Trm cells play critical roles in a rapid local immune response, which is important for preventing the invasion of pathogens [11,31]. Immunological memory is key to the success of vaccines because memory cells remember the antigen and elicit a rapid and robust immune response on re-exposure to antigens. Based on this immune memory response, a multiple-dose vaccination regimen called prime-boost enhances vaccine immunity. Maintenance of memory cells is important to confer long-term protective vaccine immunity. IL-15 controls the longevity and proliferation of memory T cells and long-lived memory B cells are generated as an output of the GC reaction [32,33].

## 3. Vaccine Development

While the T-cell response is important in determining the pathogenicity of infection, strategic induction of T-cell-mediated immunity is the key to success in vaccine development [34]. Currently, a number of candidate vaccines are evaluated in terms of the magnitude of cellular and humoral immune responses. Generally, T-cell responses are evaluated in the early phases of clinical trials, and vaccine efficacy is evaluated in terms of the protective rate, with clinical judgment in the late phase. Assessing the immunological properties of the recipients aids in determining the immunogenicity of the vaccine.

While the humoral immune response is evaluated using serological assays, the immunogenicity of vaccines is often determined in terms of the seroconversion rate of antigen-specific antibodies after vaccination. In contrast, T-cell responses are evaluated in terms of antigen-stimulated peripheral blood mononuclear cells (PBMCs). Enzyme-linked immunospot (ELISPOT) and intracellular cytokine staining are popular methods for measuring cytokine secretion from PBMCs. The evaluation of T cells producing cytokines after re-exposure to the antigen is important for understanding the effective antipathogenic immunity of vaccines. 

The appearance of variants of viruses that evade vaccines or pre-existing antiviral immunity has often raised an issue [35]. Even though booster administration of SARS-CoV-2 vaccines protects against severe disease, the newly emerging Omicron SARS-CoV-2 variant evades vaccination-induced anti-spike neutralization antibodies [36,37]. Moreover, this variant is highly resistant to therapeutic monoclonal antibodies [38]. The promotion of broadly neutralizing antibodies is effective across variants. T-cell-mediated immunity is crucial. While most current SARS-CoV-2 vaccines target spike proteins as antigens, multiple SARS-CoV-2 immunodominant T-cell epitopes are located not only in the spike but also in the M, N, and other nonstructural (NS) proteins [39]. In fact, pre-existing polymerase-specific T cells abort infection and are cross-reactive against *Coronaviridae* [26].

In DENV infection, pre-existing antibodies may increase the severity and augment viral replication [40]. Dengvaxia is the only licensed vaccine against DENV. However, it is permitted to be used only in seropositive individuals. Seronegative individuals demonstrate low vaccine efficacy, and vaccination may lead to an increased risk of severe dengue disease. Existing insufficient cross-reactive vaccine immunity exacerbates the pathogenicity of subsequent wild-type DENV infection. Dengvaxia comprises a chimeric live-attenuated yellow fever virus that expresses the structural pre-membrane (prM) and envelope (E) genes of each of the four serotypes of DENV. This chimeric vaccine includes a yellow fever NS protein. The lack of DENV NS proteins failed to induce cross-reactive T-cell responses against all serotypes of DENV [41]. DENVax (TAK-003) is a candidate vaccine for DENV that comprises a chimeric live-attenuated virus derived from the DENV2 PDK-53 strain, which was obtained by replacing the DENV-2 prM and E genes with other serotypes. DENVax has been shown to elicit durable humoral and cellular immunity and is effective against all DENV serotypes [42,43,44,45].

Target immunodominant epitopes of pathogens are also crucial for designing cross-reactive vaccines with higher immunogenicity. The pre-existence of conserved epitopes-specific CD8^+^ T cells correlates with milder disease in patients with COVID-19 [25]. Bioinformatics and peptide HLA complex analyses have revealed that epitopes derived from ORF1ab have potential as optimal vaccine antigens [46,47]. These predicted epitopes display strong immunodominant characteristics and are highly conserved.

Vaccines targeting conserved epitopes have been developed for seasonal influenza. Most commercial seasonal influenza vaccines use trivalent or quadrivalent inactivated hemagglutinin (HA) subunits as antigens. Vaccine strains are selected based on surveillance data twice a year, and the prediction of epidemic strains determines the effectiveness of influenza vaccines. An induction of T-cell responses that recognize conserved epitopes, including NS proteins, would confer multi-strain and multi-season protection. Multimeric-001/M-001 is a candidate recombinant protein vaccine that contains nine conserved B-cells, and CD4^+^ and CD8^+^ T-cell epitopes from HA, the nucleoprotein, and matrix protein 1 (M1). Flu-v is another candidate vaccine that comprises conserved peptides derived from M1, M2, and the nucleoprotein [48,49]. These two influenza vaccine candidates demonstrated a robust induction of conserved epitopes specific to cellular immunity. Unfortunately, the vaccine developer BiondVax Pharmaceuticals Ltd. recently announced that Multimeric-001/M-001 did not demonstrate significant vaccine efficacy against infections.

## 4. Technological Platforms of Vaccine

To induce cellular and humoral responses to vaccines, the development of technical platforms including antigen design, adjuvants, and delivery methods is crucial. Live-attenuated vaccines (LAVs) are traditional vaccines that have been developed as live-attenuated formulations with weakened pathogens. LAVs possess a higher immunogenic ability and induce both humoral and cellular immunity. Bacille Calmette–Guérin (BCG), which was developed in the 1920s, is regarded as the first T-cell-inducing LAV against tuberculosis [50]. BCG induces specific memory T cells that protect against the intracellular parasitic infection of *Mycobacterium tuberculosis* [51].

Inactivated or subunit vaccines have the advantage of having no risk of infection and a more stable formulation compared with LAVs [3]. Thus, inactivated antigens are used in many licensed vaccines. The administration of inactivated antigens produces antigen-specific neutralizing antibodies that protect against pathogenic infections. However, inactivated vaccines elicit a weak vaccine response compared with LAVs.

Nucleic acid-based vaccines are a relatively new technology that utilizes genetically engineered DNA/mRNA to produce an immunologic response against antigens. The ability to induce cellular and humoral responses against designed epitopes is a great advantage of these vaccines. DNA vaccines have many advantages, including safety, heat stability, ease of handling, and low cost. The concept of a DNA vaccine was introduced in the 1990s. Wolff et al. demonstrated the expression of foreign antigens by intramuscular administration of plasmid DNA [52]. The antigen expression of plasmid DNA requires delivery into the cell nucleus. Due to the limitations of delivery technology, DNA vaccines show weaker immune responses compared with other vaccine platforms. Therefore, they are often evaluated with a novel injection system for the development of DNA vaccines. ZyCoV-D was the first DNA vaccine approved in India. This SARS-CoV-2 vaccine was administered via intradermal injection combined with a jet-injector system [53].

Presently, viral vectors and nucleic acid-based vaccines are being widely used against SARS-CoV-2. Both technical platforms are based on genetic engineering that enables the induction of Th1-biased vaccine immunity by the intracellular expression of genetically engineered antigens [54,55,56,57,58,59,60,61].

## 5. Viral Vector-Based Vaccine Development

Viral vectors are a delivery tool that allows for the expression of antigens within cells. The concept of viral vectors was introduced in 1972, when Jackson et al. created recombinant DNA from the SV40 virus using genetic engineering [62]. The specific property of a vector is determined by the virus from which it is derived. In most cases, viruses are genetically engineered to reduce or eliminate pathogenicity, and most viral vectors are replication-defective.

Table 1 presents a list of the results of the representative viral vector vaccines and T-cell-mediated immunity. Depending on the target infection, several viral vectors have been used in clinical trials, and the vaccine-specific cellular and humoral immune responses have been measured. Viral vectors have received a lot of attention in the development of an HIV-1 vaccine; the induction of a virus-specific CTL response is considered essential in the case of HIV-1 because its surface envelope glycoprotein (gp120) is highly prone to mutating and evading neutralizing antibodies [63]. Although a CTL-inducing vaccine does not prevent HIV-1 infection, it has the ability to control viral loads by eliminating virus-infected cells, which slows disease progression [64]. To induce HIV-1-specific CTLs, antigens must be delivered intracellularly through the MHC class I antigen presentation pathway. Many types of recombinant viral vectors have been developed for intracellular antigen-encoded gene delivery [65].

Recombinant adenovirus (Ad) is the most suitable vector because of its high transduction efficiency, high level of transgene expression, and broad range of viral tropism. The Ad5 vector vaccine, known as MRKAd5, has received particular attention for the development of an HIV-1 vaccine. However, it was found to be unable to prevent HIV-1 infection even though it elicited CTL responses in 75% of recipients in a Phase IIb clinical trial (STEP Study/HVTN502) [66]. This study indicated that the presence of pre-existing immunity to Ad5 in vaccine recipients may not only diminish vaccine efficacy but also increase the risk of HIV-1 infection [85]. Based on this experience, circumventing anti-vector immunity and strategies for protective T-cell responses, including vaccine regimens, should be reconsidered. In 2009, a Phase III clinical trial (RV144 study) demonstrated partial protection from HIV-1 infection in Thailand [69,70,71]. The RV144 study was performed with a heterologous prime-boost regimen in which priming was carried out with an HIV-1 Env- and Gag-expressing vaccinia virus-derived vector (ALVAC-HIV (vCP1521)) and boosting was carried out with recombinant gp120 protein (AIDSVAX B/E). RV144 could induce predominant HIV-1-specific-CD4^+^ T cells. Unfortunately, RV144 exhibits modest antibody-dependent HIV-1-specific CTLs at 24%. The ALVAC-HIV/AIDSVAX B/E vaccine was halted recently because of its lack of efficacy in Phase IIb/III trials in South Africa (HVTN 702) [72].

Although the HIV-1 vaccine has not yet been licensed, several technical advances that overcome pre-existing anti-vector immunity have been developed. Ad type 26 (Ad26), type 35 (Ad35), or chimpanzee Ad (ChAd)-based vectors can circumvent anti-vector immunity due to the low seroprevalence in humans [86,87]. These vectors were used in licensed SARS-CoV-2 vaccines called Vaxzevria (ChAdOx1 nCoV-19/AZD1222), COVID-19 vaccine Janssen (Ad26.COV2.S), and Sputnik V (Gam-COVID-Vac). These vaccines induce strong CD8^+^ and Th1-dominated CD4^+^ T-cell responses [59,79,80,81,82]. Th1-biased vaccine immunity contributes to the moderate clinical outcomes of COVID-19. Furthermore, an Ad vector vaccine is expected for several other infectious diseases, such as RSV and Zika virus [83,84].

MVA is another promising vector that is derived from the highly attenuated vaccinia strain Ankara. Traditionally, the vaccinia virus has been used for smallpox vaccines, and its efficacy and safety in vaccine administration have been proven. A heterologous two-dose regimen using Ad26 (Zabdeno, Ad26 ZEBOV) and modified vaccinia Ankara (Mvabea, MVA BN-Filo) vectors has been licensed for human use against the Ebola virus disease (EVD) [77]. Although the role of T-cell-mediated immunity in EVD remains unclear, heterologous Ad26 and MVA vector vaccine regimens induce robust humoral and cellular responses that persist for 1 year after vaccination. Vesicular stomatitis virus (VSV) is also well studied as a vaccine vector. VSV has low viral pathogenicity and rare pre-existing anti-vector immunity in humans [88,89]. A VSV vector vaccine expressing the glycoprotein of an Ebola virus (Ervebo, rVSV-ZEBOV) has also been developed as a licensed EVD vaccine. Ervebo is a replication-competent viral vector vaccine that has a VSV morphology with the Ebola virus GP on its surface. Ervebo provided 100% protection against EVD in a Phase III trial that mainly targeted the production of neutralizing antibodies, even though T-cell responses to this vaccine were low to moderate [75,76].

Viral vector-based therapeutic vaccines are being developed as a therapy for HPV-associated tumors. Current licensed HPV vaccines (Gardasil, Cervarix, and Silgard) are based on virus-like particle formulations derived from expressing the capsid of HPV-L1. The induction of HPV-L1 specific humoral immunity prevents HPV infection. In contrast, eliminating infected cells is useful for treating cancerous lesions and for preventing malignant transformation of HPV-associated tumors. TA-HPV and MVA E2 are recombinant MVA vector vaccines that express the E6/E7 fusion proteins and E2 protein of HPV, respectively [73,74]. The induction of cellular immunity directed against the oncogene products E2, E6, and E7 would be effective for cancer therapeutic vaccines.

## 6. mRNA Vaccine Development

The concept of mRNA vaccines was proposed in the 1990s [52]. The immunogenicity of these vaccines is determined by the translation of antigen-encoded mRNA into cells. mRNA is a small molecule that allows for repeated administration, avoiding anti-vector immunity. Moreover, it is safe and has no risk of infection or insertional mutagenesis as the translation of mRNA occurs in the ribosome. The first animal study involving an mRNA vaccine demonstrated an anti-influenza CD8^+^ T-cell response in mice [90]. mRNA vaccines have the advantage of rapid and low-cost manufacturing processes compared with other vaccine platforms. They can produce an in vitro transcription enzyme reaction with a cell-free manufacturing process and no animal-derived raw materials. Thus, mRNA can omit time-consuming processes involved in conventional vaccine manufacturing [91,92].

Table 2 summarizes the results of the representative mRNA vaccines and T-cell-mediated immunity. During the COVID-19 pandemic, two SARS-CoV-2 vaccines, mRNA-1273 and tozinameran (BNT162b2), were licensed for human use as mRNA vaccines for the first time. Both vaccines induced both neutralizing antibodies and Th1-biased SARS-CoV-2 specific T-cell responses with high vaccine efficacy in Phase III clinical trials [93,94]. In pre-clinical studies, an increase in Tfh cells in draining lymph nodes was also observed, which may confer long-term protective antibody responses [55,95].

Instability and translation efficacy are two major issues in the clinical use of mRNA vaccines. mRNA modification and nanomaterial encapsulation are two strategies that help address these issues and are, thus, commonly used in mRNA vaccines.

### 6.1. mRNA Modification

mRNA modifications can improve the stability of mRNA vaccines. Major mRNA modification strategies include the following: (1) adding a 5′ cap analog, (2) optimizing 5′ and 3′ untranslated regions (UTRs), (3) elongating the poly(A) tail, (4) optimizing the codon in the open reading frame, and (5) modifying the nucleoside by substituting uridine with pseudouridine [101,102]. Adding a cap analog at the 5′ end of mRNA enhances its stability and improves translation efficacy [103,104]. The 5′ capping of mRNA prevents degradation by exonuclease and promotes binding to the eukaryotic translation initiation factor 4E [105,106]. Regulatory elements in the 5′ UTR and the length of the 3′-UTR also increase translation efficacy through reactions with RNA-binding proteins [107]. The length of the poly(A) tail stabilizes mRNA and is closely associated with translation efficiency [108,109]. Selecting an optimized codon also improves the rate of antigen translation. It involves the secondary structure of mRNA, mRNA stability, and the translation elongation rate [110,111]. Replacing appropriate synonymous codons and GC-rich sequences in mRNA increases translation efficiency [112]. In addition, the modified nucleoside enhances protein expression and reduces immunogenicity in mammalian cells [113]. The replacement of uridine with N1-methyl-pseudouridine (m1Ψ) is the most popular modification and has been adopted in mRNA vaccine design [114].

### 6.2. mRNA Encapsulation

The encapsulation of mRNAs within designed nanomaterials is a common method used for developing mRNA vaccines. Since mRNA is located in the core of the nanoparticles (NPs), encapsulation allows for protection from nuclease degradation in vivo and improves the chemical stability, hydrolysis, and oxidation during storage [115]. Moreover, appropriate nanomaterials also enable the delivery of mRNA to target immune cells. Naked mRNA has a highly negative charge density, and cationic materials help fuse with host cells and improve the in vivo stability of the mRNA.

Numerous polymeric materials (chitosan, polyethylenamine, PLGA, and γ-PGA), lipids (DOSPA, DOPE, and DOTAP), and proteins (protamine) have been investigated for encapsulation of vaccine antigens [116,117]. Among these, LNPs are the most popular technology for mRNA vaccines. Typical LNPs comprise four lipid components: cationic lipids, cholesterol, phospholipids, and polyethylene glycol (PEG). Cationic lipids facilitate the intracellular delivery of mRNA by host cell membrane fusion during internalization. They also enhance the charge-driven encapsulation with negatively charged mRNAs during the manufacturing process. Phospholipids enhance stability through the conformation of bilayered lipid structures. PEG extends the half-life of LNPs, which controls the particle size and prevents aggregation during storage.

The particle size of LNP-mRNA is approximately 60–100 nm upon mixing during production. The size and shape of LNP-mRNA are associated with the in vivo durability, distribution, and immunogenicity of the vaccine [118]. The administration of NPs <100 nm in size is likely to drain the lymph nodes, which enhances the immunogenicity of the vaccine. NP administration induces transient inflammation that drives the recruitment of neutrophils and APCs, and the designed nanomaterials act as adjuvants and are useful for the induction of appropriate vaccine immunity [10].

## 7. Target Delivery of a Vaccine

Most licensed vaccines are administered intramuscularly. The delivery route of antigens affects immunogenicity due to the presence of tissue-resident immune cells. This indicates that an alternate administration route has the potential to improve the immunological properties of a vaccine [119]. Moreover, the generation of specific Trm cells is important because it enables a rapid response upon re-exposure to the antigen [11]. Therefore, the delivery route of vaccines and the mediation of local inflammation should be considered with novel medical devices and biomaterials.

Intradermal (ID) administration enhances vaccine immunogenicity and provides dose-sparing effects in the case of seasonal influenza vaccines [120]. ID delivery stimulates resident immune cells that rapidly enhance humoral and cellular responses. The ID route is occasionally used in nucleic acid-based DNA and mRNA vaccines with novel medical devices [121]. ID vaccination with mRNA-LNPs encoding various viral surface antigens induces a strong antigen-specific Tfh cell response associated with long-living/high-affinity neutralizing antibodies and durable protection [122]. A thermostable microneedle vaccine patch is advantageous owing to less patient pain, enhanced immunogenicity, and possible self-administration [123].

Intranasal administration can induce systemic and local mucosal immunity [124]. Mucosal immunity blocks respiratory pathogen invasion by producing IgA at the mucosal surface. Moreover, the use of a noninvasive, needle-free nasal route is advantageous for vaccination. An intranasal, live-attenuated influenza vaccine is available for use [125].

Moreover, several functional biomaterials have been developed to enhance the immunogenicity of vaccines. Scaffold-based vaccines utilize pore-forming polymer gel matrices combined with immune modulating components such as adjuvants and cytokines to concentrate and stimulate immune cells at the site of injection [126]. Besides this, some functional materials conjugated with an antigen have also demonstrated effective delivery to the lymph nodes [127].

## 8. Conclusions and Future Directions

The induction of T-cell-mediated responses is key to vaccine development. The results of clinical outcome studies of many infectious diseases show that the most effective anti-pathogen immunity induces both cellular and humoral immune responses. Elucidating the antipathogenic immune response clarifies the mechanism of action of effective vaccines. Currently, the assessment of T-cell responses is common in the early phases of clinical trials. The characteristics of specific vaccine-induced T cells indicate the potential of these vaccines. Th-cell differentiation regulates the bias towards humoral or cellular immunity. The presence of cross-reactive CTLs reduces the risk of ADEs. Memory cells are associated with the duration of vaccine immunity. In nonclinical or clinical trials of vaccines, criteria should be set based on the role of T cells. Vaccine efficacy and immunological response vary between individuals; older adults exhibit delayed and reduced humoral and cellular responses compared with young adults. Aging shows a bias of differentiating into short-lived effector T cells rather than Tfh cells or memory cells [128]. Therefore, the immunogenicity of a vaccine needs to be assessed in several age groups, including high-risk populations, for targeted infectious diseases. Because of the weak immune response in people who are immunocompromised or older, it may be essential to use booster doses for these populations to elicit vaccine immunity. 

Many technical platforms, including targeted cell delivery of antigens, have been developed to induce antipathogenic immunity. Viral vectors enable intracellular expression of foreign-encoded genes and confer a robust Th1-dominant response without any adjuvant. The feature of a vector depends on its viral tropism, which enables the delivery of foreign genes to target cells. Viral vectors are well tolerated and are already being used in some licensed vaccines despite the disadvantage of anti-vector immunity. Viral vectors are also regarded as useful delivery platforms for cell and gene therapy, including genome editing. Adeno-associated viral and lentiviral vectors are widely used for gene therapy [129]. These vectors can infect both dividing and nondividing cells and provide long-term expression of foreign genes.

Recently, mRNA-based SARS-CoV-2 vaccines have attracted attention. mRNA modification and LNP technologies help to address the instability of mRNAs. Based on the results of COVID-19 vaccines, the mRNA vaccines are potentially well tolerated and effective. mRNA vaccines can be produced by rapid, inexpensive, and scalable manufacturing methods. Therefore, the development of LNP-mRNA vaccines against other infectious diseases, such as influenza virus, RSV, and Zika virus, is accelerating [57,99,130,131]. Self-amplifying mRNA (saRNA) is being developed as a next-generation technology for mRNA vaccines. saRNA encodes four NS proteins, NSP1–4, which are derived from alphaviruses. NSP1–4 encode a replicase involved in the amplification of saRNA, and self-replicative activity allows for low doses [56]. Trans-amplifying mRNA is a further advanced technology that was developed for the lowest dose of vaccines [132]. Continuous technology updates definitely help to provide a rapid emergency response against the next pandemic [92].

LNPs are a popular delivery system for mRNA. Nanomaterial encapsulation is also useful as a protein or peptide antigen. An advantage of NPs is that they extend the persistence of an antigen at the injection site, which enhances immunogenicity. Moreover, NP vaccines confer durable humoral immunity by enhancing Tfh cells and by promoting germinal center induction [133].

Technical platforms of nucleic acid-based vaccines have also become a promising platform for cancer immunotherapy. Clinical trials of several mRNA vaccines encoding tumor-associated antigens are ongoing. In addition, delivering mRNA encoding immune-modulating genes has the potential to reshape the tumor microenvironment [134]. Furthermore, lentiviral or retroviral vectors are used in the manufacture of chimeric antigen receptor (CAR)-T cells, which are effective for cancer immunotherapy [135].

Preferable vaccine immunity is not the same in infectious diseases, and strategic T-cell induction is desirable for effective vaccines. Although the role of T cells has not been fully characterized, multiple functions of T cells have been elucidated, including moderate severity of illness, control of the viral load, elimination of infected cells, and protection from infection. These advantageous features of T cells further support ongoing efforts to develop strategic T-cell-inducing vaccines.

## Figures and Tables

**Table 1:** List of results of representative viral vector vaccines and T cell-mediated immunity.

Target	Vaccine Name	Status	Platform Technology	Results	References
HIV-1	MEKAd5	Phase II (STEP Study/HVTN502)	Recombinant Ad5 vector encoding Gag/Pol/Nef genes of HIV-1	Did not prevent HIV-1 infection although IFN-γ ELISPOT response was observed in 75% of recipients.	[66]
		Phase IIb (Phambili Study/HVTN 503)		Did not prevent HIV-1 infection although IFN-γ ELISPOT responses to Clade B and Clade C peptides were observed in 89.2% and 77.4% of recipients, respectively.	[67,68]
	ALVAC-HIV (vCP1521) AIDSVAX B/E	Phase III (RV144)	Heterologous prime-boost regimen: Prime: ALVAC vector encoding Env/Gag/Pol of HIV-1 Boost: recombinant Env gp120 protein combined with alum adjuvant	Reduced the risk of HIV infection. Vaccine could induce predominant HIV-1-specific CD4^+^ T cells. CTLs were observed in 24% of recipients.	[69,70,71]
	ALVAC-HIV (vCP2438) AIDSVAX B/E	Phase IIb/III (HVTN 702)	Heterologous prime-boost regimen: Prime: ALVAC vector encoding Env/Gag/Pol of HIV-1 Boost: recombinant Env gp120 protein combined with MF59 adjuvant	Did not prevent HIV-1 infection. CD4^+^ and CD8^+^ T cells responses were observed.	[72]
HPV	TA-HPV	Phase II	Recombinant MVA vector expressing E6/E7 fusion proteins from HPV-16 and HPV-18	Decrease in lesion size by at least 40% observed in 83% of patients. HPV-specific CTL responses were observed.	[73]
	MVA E2	Phase III	Recombinant MVA vector expressing E2 protein of HPV	89.3% efficacy in the elimination of HPV-induced intraepithelial lesions. HPV-specific CTL responses were observed.	[74]
Ebola virus	Ervebo (rVSV-ZEBOV)	Licensed in 2019	A live, replication-competent vector in which the VSV glycoprotein (G) gene is replaced with the glycoprotein (GP) gene of ZEBOV.	100% protection against Ebola virus disease. Low to moderate specific T cell responses were observed in clinical trials but had the potency to induce antigen-specific T cell responses.	[75,76]
	Zabdeno (Ad26.ZEBOV) Mvabea (MVA-BN-Filo)	Licensed in 2020	Heterologous prime-boost regimen: Prime: glycoprotein expressing rAd26 vector Boost: glycoprotein- and nucleoprotein-expressing MVA vector	Vaccine induced humoral and CD4^+^ and CD8^+^ T cell responses that persisted for 1 year post vaccination.	[77]
	ChAd3-EBO-Z	Phase III	Recombinant ChAd vector encoding ebolavirus glycoprotein genes	Vaccine induced dose-dependent CD4^+^ and CD8^+^ T cell responses.	[78]
SARS-CoV-2	COVID-19 vaccine Janssen (Ad26.COV2.S)	Licensed in 2020	Recombinant Ad26 vector encoding spike genes from SARS-CoV-2	Vaccine efficacy 85% and 66% against severe/moderate to severe/critical COVID-19, respectively.	[59,79]
	Vaxzevria (ChAdOx1 nCoV-19/AZD1222)	Licensed in 2020	Recombinant ChAd vector encoding spike genes from SARS-CoV-2	Vaccine efficacy 76.0% and 82.4% after first and second dose of vaccination, respectively. Vaccination induced Th1-biased CD4^+^ and CD8^+^ T cell responses.	[80,81,82]
	Sputnik V (Gam-COVID-Vac)	Licensed in 2020	Heterologous prime-boost regimen: Prime: spike protein expressing rAd26 vector Boost: spike protein expressing rAd5 vector	Vaccine efficacy was 91.6%. Vaccine induced robust humoral and cellular immune responses. Higher level of IFN-γ secreting PBMCs was observed.	[60]
RSV	Ad26.RSV.preF	Phase III	Recombinant Ad26 vector encoding prefusion F genes from RSV	Vaccine induced a high neutralizing antibody titer and long-lasting Th1-biased immunity.	[83]
ZIKV	Ad26.ZIKV.001	Phase I	Recombinant Ad26 vector encoding ZIKV M-Env	Vaccine induced humoral immune response and antibody titers persisting for at least 1 year. Env-specific cellular responses were induced.	[84]

Abbreviations: CTLs, cytotoxic T lymphocytes; ELISPOT, enzyme-linked immunospot; HPV, human papilloma virus; MVA, modified vaccinia ankara; VSV, vesicular stomatitis virus; ZEBOV, Ebola virus zaire; Ad26, adenovirus type 26; ChAd, chimpanzee adenovirus; RSV, respiratory syncytial virus; ADE, antibody-dependent enhancement; ZIKV, Zika virus; PBMCs, peripheral blood mononuclear cells; IFN, interferon.

**Table 2:** List of results of representative mRNA vaccines and T-cell-mediated immunity.

Target	Vaccine Name	Status	Platform Technology	Results	References
SARS-CoV-2	Tozinameran (BNT162b2)	Licensed in 2020	LNP-mRNA encoding full length S protein of SARS-CoV-2	95% effective against COVID-19. Vaccination induces 94.4% and 80.6% IFN-γ-producing CD4^+^ and CD8^+^ T cells, respectively. Increased Th1-biased Tfh cells have been observed in draining lymph node in pre-clinical studies.	[54,93,95,96]
	mRNA-1273	Licensed in 2020	LNP-mRNA encoding receptor binding domain of S protein	94.1% efficacy at preventing COVID-19 illness, including severe disease. Vaccination induces Th1-biased CD4^+^ T cell response and low CD8^+^ T cell responses. IL-21-secreting Tfh cell response also observed in pre-clinical studies.	[55,94,97,98]
	COVAC1 (LNP-nCoVsaRNA)	Phase I	LNP-self-amplifying mRNA encodes an RNA replicase derived from an alphavirus and SARS-CoV-2 prefusion stabilized S protein.	Robust humoral- and Th1-biased cellular responses observed in mice.	[56]
Endemic avian H10N8 and H7N9 influenza viruses	mRNA-1851	Phase I	LNP-mRNA encoding the full-length, membrane-bound form of hemagglutinin from the H10N8 or H7N9 influenza strain.	Well-tolerated and robust humoral immune responses observed. However, HA-specific cell-mediated responses were not detected by IFN-γ ELISPOT	[99]
Seasonal influenza	mRNA-1010/MRT5400/MRT5401	Phase I/II	LNP-mRNA encoding the influenza HA protein	NA	[100]
RSV	mRNA-1172/mRNA-1345/mRNA-1777 (V171)	Phase I	LNP-mRNA encoding the RSV prefusion F protein	Robust CD4^+^ and CD8^+^ T-cell responses in mice.	[57]
ZIKV	mRNA-1893	Phase I	Encoding the structural proteins of the Zika virus. Designed to cause cells to secrete virus-like particles.	In the flavivirus-seronegative group, seroconversion rates after the second vaccination reached 94.4% at the lower dose and 100% at the higher dose.	Clinical Trials NCT04917861

Abbreviations: LNPs, lipid nanoparticles; Tfh, T follicular helper; ELISPOT, enzyme-linked immunospot; RSV, respiratory syncytial virus; ADE, antibody-dependent enhancement; DENV, dengue virus; ZIKV, Zika virus; IFN, interferon.

## References

[1] Fazilleau N., Mark L., McHeyzer-Williams L.J., McHeyzer-Williams M.G. (2009). Follicular helper T cells: Lineage and location. Immunity.

[2] Crotty S. (2014). T follicular helper cell differentiation, function, and roles in disease. Immunity.

[3] Pollard A.J., Bijker E.M. (2021). A guide to vaccinology: From basic principles to new developments. Nat. Rev. Immunol..

[4] Avci F., Berti F., Dull P., Hennessey J., Pavliak V., Prasad A.K., Vann W., Wacker M., Marcq O. (2019). Glycoconjugates: What It Would Take To Master These Well-Known yet Little-Understood Immunogens for Vaccine Development. mSphere.

[5] Wild K., Smits M., Killmer S., Strohmeier S., Neumann-Haefelin C., Bengsch B., Krammer F., Schwemmle M., Hofmann M., Thimme R. (2021). Pre-existing immunity and vaccine history determine hemagglutinin-specific CD4 T cell and IgG response following seasonal influenza vaccination. Nat. Commun..

[6] Sridhar S., Begom S., Bermingham A., Hoschler K., Adamson W., Carman W., Bean T., Barclay W., Deeks J., Lalvani A. (2013). Cellular immune correlates of protection against symptomatic pandemic influenza. Nat. Med..

[7] Wilkinson T.M., Li C.K.F., Chui C.S.C., Huang A.K.Y., Perkins M., Liebner J.C., Lambkin-Williams R., Gilbert A.S., Oxford J., Nicholas B. (2012). Preexisting influenza-specific CD4^+^ T cells correlate with disease protection against influenza challenge in humans. Nat. Med..

[8] Graham B.S. (2020). Rapid COVID-19 vaccine development. Science.

[9] Hokello J., Sharma A.L., Tyagi M. (2021). An Update on the HIV DNA Vaccine Strategy. Vaccines.

[10] Fries C.N., Curvino E.J., Chen J.L., Permar S.R., Fouda G.G., Collier J.H. (2021). Advances in nanomaterial vaccine strategies to address infectious diseases impacting global health. Nat. Nanotechnol..

[11] Schenkel J.M., Masopust D. (2014). Tissue-resident memory T cells. Immunity.

[12] Yusuf H., Kett V. (2017). Current prospects and future challenges for nasal vaccine delivery. Hum. Vaccin Immunother..

[13] Menon I., Bagwe P., Gomes K., Bajaj L., Gala R., Uddin M., D’Souza M., Zughaier S. (2021). Microneedles: A New Generation Vaccine Delivery System. Micromachines.

[14] Rosendahl Huber S., van Beek J., de Jonge J., Luytjes W., van Baarle D. (2014). T cell responses to viral infections—Opportunities for Peptide vaccination. Front. Immunol..

[15] van der Burg S.H., Melief C.J. (2011). Therapeutic vaccination against human papilloma virus induced malignancies. Curr. Opin. Immunol..

[16] Davenport M.P., Petravic J. (2010). CD8^+^ T cell control of HIV-a known unknown. PLoS Pathog..

[17] Harty J.T., Tvinnereim A.R., White D.W. (2000). CD8^+^ T cell effector mechanisms in resistance to infection. Annu. Rev. Immunol..

[18] Prabhudas M., Bonney E., Caron K., Dey S., Erlebacher A., Fazleabas A., Fisher S., Golos T., Matzuk M., McCune J.M. (2015). Immune mechanisms at the maternal-fetal interface: Perspectives and challenges. Nat. Immunol..

[19] Guthmiller J.J., Stovicek O., Wang J., Changrob S., Li L., Halfmann P., Zheng N.Y., Utset H., Stamper C.T., Dugan H.L. (2021). SARS-CoV-2 Infection Severity Is Linked to Superior Humoral Immunity against the Spike. MBio.

[20] Chen G., Wu D., Guo W., Cao Y., Huang D., Wang H., Wang T., Zhang X., Chen H., Yu H. (2020). Clinical and immunological features of severe and moderate coronavirus disease 2019. J. Clin. Invest..

[21] Leung S., Liu X., Fang L., Chen X., Guo T., Zhang J. (2010). The cytokine milieu in the interplay of pathogenic Th1/Th17 cells and regulatory T cells in autoimmune disease. Cell Mol. Immunol..

[22] Khader S.A., Gaffen S.L., Kolls J.K. (2009). Th17 cells at the crossroads of innate and adaptive immunity against infectious diseases at the mucosa. Mucosal. Immunol..

[23] Ndure J., Flanagan K.L. (2014). Targeting regulatory T cells to improve vaccine immunogenicity in early life. Front. Microbiol..

[24] Loyal L., Braun J., Henze L., Kruse B., Dingeldey M., Reimer U., Kern F., Schwarz T., Mangold M., Unger C. (2021). Cross-reactive CD4^+^ T cells enhance SARS-CoV-2 immune responses upon infection and vaccination. Science..

[25] Mallajosyula V., Ganjavi C., Chakraborty S., McSween A.M., Pavlovitch-Bedzyk A.J., Wilhelmy J., Nau A., Manohar M., Nadeau K.C., Davis M.M. (2021). CD8^+^ T cells specific for conserved coronavirus epitopes correlate with milder disease in COVID-19 patients. Sci. Immunol..

[26] Swadling L., Diniz M.O., Schmidt N.M., Amin O.E., Chandran A., Shaw E., Pade C., Gibbons J.M., Le Bert N., Tan A.T. (2022). Pre-existing polymerase-specific T cells expand in abortive seronegative SARS-CoV-2. Nature.

[27] Le Bert N., Clapham H.E., Tan A.T., Chia W.N., Tham C.Y.L., Lim J.M., Kunasegaran K., Tan L., Dutertre C.A., Shankar N. (2021). Highly functional virus-specific cellular immune response in asymptomatic SARS-CoV-2 infection. J. Exp. Med..

[28] Le Bert N., Tan A.T., Kunasegaran K., Tham C.Y.L., Hafezi M., Chia A., Chng M., Lin M., Tan N., Linster M. (2020). SARS-CoV-2-specific T cell immunity in cases of COVID-19 and SARS, and uninfected controls. Nature.

[29] Grifoni A., Weiskopf D., Ramirez S.I., Mateus J., Dan J.M., Moderbacher C.R., Rawlings S.A., Sutherland A., Premkumar L., Jadi R.S. (2020). Targets of T Cell Responses to SARS-CoV-2 Coronavirus in Humans with COVID-19 Disease and Unexposed Individuals. Cell.

[30] Woodland D.L., Kohlmeier J.E. (2009). Migration, maintenance and recall of memory T cells in peripheral tissues. Nat. Rev. Immunol..

[31] Park C.O., Kupper T.S. (2015). The emerging role of resident memory T cells in protective immunity and inflammatory disease. Nat. Med..

[32] Nolz J.C., Richer M.J. (2020). Control of memory CD8^+^ T cell longevity and effector functions by IL-15. Mol. Immunol..

[33] Zhang Y., Garcia-Ibanez L., Ulbricht C., Lok L.S.C., Pike J.A., Mueller-Winkler J., Dennison T.W., Ferdinand J.R., Burnett C.J.M., Yam-Puc J.C. (2022). Recycling of memory B cells between germinal center and lymph node subcapsular sinus supports affinity maturation to antigenic drift. Nat. Commun..

[34] Painter M.M., Mathew D., Goel R.R., Apostolidis S.A., Pattekar A., Kuthuru O., Baxter A.E., Herati R.S., Oldridge D.A., Gouma S. (2021). Rapid induction of antigen-specific CD4^+^ T cells is associated with coordinated humoral and cellular immunity to SARS-CoV-2 mRNA vaccination. Immunity.

[35] Krause P.R., Fleming T.R., Longini I.M., Peto R., Briand S., Heymann D.L., Beral V., Snape M.D., Rees H., Ropero A.M. (2021). SARS-CoV-2 Variants and Vaccines. N. Engl. J. Med..

[36] Accorsi E.K., Britton A., Fleming-Dutra K.E., Smith Z.R., Shang N., Derado G., Miller J., Schrag S.J., Verani J.R. (2022). Association Between 3 Doses of mRNA COVID-19 Vaccine and Symptomatic Infection Caused by the SARS-CoV-2 Omicron and Delta Variants. JAMA.

[37] Collie S., Champion J., Moultrie H., Bekker L.G., Gray G. (2021). Effectiveness of BNT162b2 Vaccine against Omicron Variant in South Africa. N. Engl. J. Med..

[38] Hoffmann M., Krüger N., Schulz S., Cossmann A., Rocha C., Kempf A., Nehlmeier I., Graichen L., Moldenhauer A.-S., Winkler M.S. (2021). The Omicron variant is highly resistant against antibody-mediated neutralization: Implications for control of the COVID-19 pandemic. Cell.

[39] Tarke A., Sidney J., Kidd C.K., Dan J.M., Ramirez S.I., Yu E.D., Mateus J., da Silva Antunes R., Moore E., Rubiro P. (2021). Comprehensive analysis of T cell immunodominance and immunoprevalence of SARS-CoV-2 epitopes in COVID-19 cases. Cell Rep. Med..

[40] St John A.L., Rathore A.P.S. (2019). Adaptive immune responses to primary and secondary dengue virus infections. Nat. Rev. Immunol..

[41] Martinez D.R., Yount B., Nivarthi U., Munt J.E., Delacruz M.J., Whitehead S.S., Durbin A.P., de Silva A.M., Baric R.S. (2020). Antigenic Variation of the Dengue Virus 2 Genotypes Impacts the Neutralization Activity of Human Antibodies in Vaccinees. Cell Rep..

[42] Saez-Llorens X., Tricou V., Yu D., Rivera L., Jimeno J., Villarreal A.C., Dato E., Mazara S., Vargas M., Brose M. (2018). Immunogenicity and safety of one versus two doses of tetravalent dengue vaccine in healthy children aged 2–17 years in Asia and Latin America: 18-month interim data from a phase 2, randomised, placebo-controlled study. Lancet Infect. Dis..

[43] Biswal S., Reynales H., Saez-Llorens X., Lopez P., Borja-Tabora C., Kosalaraksa P., Sirivichayakul C., Watanaveeradej V., Rivera L., Espinoza F. (2019). Efficacy of a Tetravalent Dengue Vaccine in Healthy Children and Adolescents. N. Engl. J. Med..

[44] Waickman A.T., Friberg H., Gargulak M., Kong A., Polhemus M., Endy T., Thomas S.J., Jarman R.G., Currier J.R. (2019). Assessing the Diversity and Stability of Cellular Immunity Generated in Response to the Candidate Live-Attenuated Dengue Virus Vaccine TAK-003. Front. Immunol..

[45] Waickman A.T., Victor K., Li T., Hatch K., Rutvisuttinunt W., Medin C., Gabriel B., Jarman R.G., Friberg H., Currier J.R. (2019). Dissecting the heterogeneity of DENV vaccine-elicited cellular immunity using single-cell RNA sequencing and metabolic profiling. Nat. Commun..

[46] Gustiananda M., Sulistyo B.P., Agustriawan D., Andarini S. (2021). Immunoinformatics Analysis of SARS-CoV-2 ORF1ab Polyproteins to Identify Promiscuous and Highly Conserved T-Cell Epitopes to Formulate Vaccine for Indonesia and the World Population. Vaccines.

[47] Gangaev A., Ketelaars S.L.C., Isaeva O.I., Patiwael S., Dopler A., Hoefakker K., Biasi S.D., Gibellini L., Mussini C., Guaraldi G. (2021). Identification and characterization of a SARS-CoV-2 specific CD8^+^ T cell response with immunodominant features. Nat. Commun..

[48] Pleguezuelos O., James E., Fernandez A., Lopes V., Rosas L.A., Cervantes-Medina A., Cleath J., Edwards K., Neitzey D., Gu W. (2020). Efficacy of FLU-v, a broad-spectrum influenza vaccine, in a randomized phase IIb human influenza challenge study. NPJ Vaccines.

[49] Atsmon J., Kate-Ilovitz E., Shaikevich D., Singer Y., Volokhov I., Haim K.Y., Ben-Yedidia T. (2012). Safety and immunogenicity of multimeric-001—A novel universal influenza vaccine. J. Clin. Immunol..

[50] Gilbert S.C. (2012). T-cell-inducing vaccines—What’s the future. Immunology.

[51] Colditz G.A., Brewer T.F., Berkey C.S., Wilson M.E., Burdick E., Fineberg H.V., Mosteller F. (1994). Efficacy of BCG vaccine in the prevention of tuberculosis. Meta-analysis of the published literature. JAMA.

[52] Wolff J.A., Malone R.W., Williams P., Chong W., Acsadi G., Jani A., Felgner P.L. (1990). Direct gene transfer into mouse muscle in vivo. Science.

[53] Sheridan C. (2021). First COVID-19 DNA vaccine approved, others in hot pursuit. Nat. Biotechnol..

[54] Sahin U., Muik A., Derhovanessian E., Vogler I., Kranz L.M., Vormehr M., Baum A., Pascal K., Quandt J., Maurus D. (2020). COVID-19 vaccine BNT162b1 elicits human antibody and TH1 T cell responses. Nature.

[55] Corbett K.S., Flynn B., Foulds K.E., Francica J.R., Boyoglu-Barnum S., Werner A.P., Flach B., O’Connell S., Bock K.W., Minai M. (2020). Evaluation of the mRNA-1273 Vaccine against SARS-CoV-2 in Nonhuman Primates. N. Engl. J. Med..

[56] McKay P.F., Hu K., Blakney A.K., Samnuan K., Brown J.C., Penn R., Zhou J., Bouton C.R., Rogers P., Polra K. (2020). Self-amplifying RNA SARS-CoV-2 lipid nanoparticle vaccine candidate induces high neutralizing antibody titers in mice. Nat. Commun..

[57] Aliprantis A.O., Shaw C.A., Griffin P., Farinola N., Railkar R.A., Cao X., Liu W., Sachs J.R., Swenson C.J., Lee H. (2021). A phase 1, randomized, placebo-controlled study to evaluate the safety and immunogenicity of an mRNA-based RSV prefusion F protein vaccine in healthy younger and older adults. Hum. Vaccin. Immunother..

[58] Bos R., Rutten L., van der Lubbe J.E.M., Bakkers M.J.G., Hardenberg G., Wegmann F., Zuijdgeest D., Wilde A.H.d., Koornneef A., Verwilligen A. (2020). Ad26 vector-based COVID-19 vaccine encoding a prefusion-stabilized SARS-CoV-2 Spike immunogen induces potent humoral and cellular immune responses. NPJ Vaccines.

[59] Sadoff J., Le Gars M., Shukarev G., Heerwegh D., Truyers C., de Groot A.M., Stoop J., Tete S., Damme W.V., Leroux-Roels I. (2021). Interim Results of a Phase 1–2a Trial of Ad26.COV2.S COVID-19 Vaccine. N. Engl. J. Med..

[60] Logunov D.Y., Dolzhikova I.V., Shcheblyakov D.V., Tukhvatulin A.I., Zubkova O.V., Dzharullaeva A.S., Kovyrshina A.V., Lubenets N.L., Grousova D.M., Erokhova A.S. (2021). Safety and efficacy of an rAd26 and rAd5 vector-based heterologous prime-boost COVID-19 vaccine: An interim analysis of a randomised controlled phase 3 trial in Russia. Lancet.

[61] Momin T., Kansagra K., Patel H., Sharma S., Sharma B., Patel J., Mittal R., Sanmukhani J., Maithal K., Dey A. (2021). Safety and Immunogenicity of a DNA SARS-CoV-2 vaccine (ZyCoV-D): Results of an open-label, non-randomized phase I part of phase I/II clinical study by intradermal route in healthy subjects in India. EClinicalMedicine.

[62] Jackson D.A., Symons R.H., Berg P. (1972). Biochemical method for inserting new genetic information into DNA of Simian Virus 40: Circular SV40 DNA molecules containing lambda phage genes and the galactose operon of *Escherichia coli*. Proc. Natl. Acad. Sci. USA.

[63] Kalidasan V., Theva Das K. (2020). Lessons Learned From Failures and Success Stories of HIV Breakthroughs: Are We Getting Closer to an HIV Cure?. Front. Microbiol..

[64] McMichael A.J. (2006). HIV vaccines. Annu. Rev. Immunol..

[65] Ura T., Okuda K., Shimada M. (2014). Developments in Viral Vector-Based Vaccines. Vaccines.

[66] Buchbinder S.P., Mehrotra D.V., Duerr A., Fitzgerald D.W., Mogg R., Li D., Gilbert P.B., Lama J.R., Marmor M., Rio C.D. (2008). Efficacy assessment of a cell-mediated immunity HIV-1 vaccine (the Step Study): A double-blind, randomised, placebo-controlled, test-of-concept trial. Lancet.

[67] Gray G.E., Allen M., Moodie Z., Churchyard G., Bekker L.G., Nchabeleng M., Mlisana M., Metch B., Bruyn G.d., Latka M.H. (2011). Safety and efficacy of the HVTN 503/Phambili study of a clade-B-based HIV-1 vaccine in South Africa: A double-blind, randomised, placebo-controlled test-of-concept phase 2b study. Lancet Infect. Dis..

[68] Gray G.E., Moodie Z., Metch B., Gilbert P.B., Bekker L.G., Churchyard G., Nchabeleng M., Mlisana K., Laher F., Roux S. (2014). Recombinant adenovirus type 5 HIV gag/pol/nef vaccine in South Africa: Unblinded, long-term follow-up of the phase 2b HVTN 503/Phambili study. Lancet Infect. Dis..

[69] Rerks-Ngarm S., Pitisuttithum P., Nitayaphan S., Kaewkungwal J., Chiu J., Paris R., Premsri N., Namwat C., De Souza M., Adams E. (2009). Vaccination with ALVAC and AIDSVAX to prevent HIV-1 infection in Thailand. N. Engl. J. Med..

[70] Kim J.H., Excler J.L., Michael N.L. (2015). Lessons from the RV144 Thai phase III HIV-1 vaccine trial and the search for correlates of protection. Annu. Rev. Med..

[71] De Souza M.S., Ratto-Kim S., Chuenarom W., Schuetz A., Chantakulkij S., Nuntapinit B., Valencia-Micolta A., Thelian D., Nitayaphan S., Pitisuttithum P. (2012). The Thai phase III trial (RV144) vaccine regimen induces T cell responses that preferentially target epitopes within the V2 region of HIV-1 envelope. J. Immunol..

[72] Gray G.E., Bekker L.-G., Laher F., Malahleha M., Allen M., Moodie Z., Grunenberg N., Huang Y., Grove D., Prigmore B. (2021). Vaccine Efficacy of ALVAC-HIV and Bivalent Subtype C gp120-MF59 in Adults. N. Engl. J. Med..

[73] Baldwin P.J., Van Der Burg S.H., Boswell C.M., Offringa R., Hickling J.K., Dobson J., Roberts J.S.C., A Latimer J., Moseley R.P., Coleman N. (2003). Vaccinia-expressed human papillomavirus 16 and 18 e6 and e7 as a therapeutic vaccination for vulval and vaginal intraepithelial neoplasia. Clin. Cancer Res..

[74] Rosales R., López-Contreras M., Rosales C., Magallanes-Molina J.-R., Gonzalez-Vergara R., Arroyo-Cazarez J.M., Ricardez-Arenas A., Del Follo-Valencia A., Padilla-Arriaga S., Guerrero M.V. (2014). Regression of human papillomavirus intraepithelial lesions is induced by MVA E2 therapeutic vaccine. Hum. Gene Ther..

[75] Monath T.P., Fast P.E., Modjarrad K., Clarke D.K., Martin B.K., Fusco J., Nichols R., Heppner D.G., Simon J.K., Dubey S. (2019). rVSVDeltaG-ZEBOV-GP (also designated V920) recombinant vesicular stomatitis virus pseudotyped with Ebola Zaire Glycoprotein: Standardized template with key considerations for a risk/benefit assessment. Vacccine.

[76] Dahlke C., Kasonta R., Lunemann S., Krahling V., Zinser M.E., Biedenkopf N., Fehling S.K., Ly M.L., Rechtien A., Stubbe H.C. (2017). Dose-dependent T-cell Dynamics and Cytokine Cascade Following rVSV-ZEBOV Immunization. EBioMedicine.

[77] Pollard A.J., Launay O., Lelievre J.-D., Lacabaratz C., Grande S., Goldstein N., Robinson C., Gaddah A., Bockstal V., Wiedemann A. (2021). Safety and immunogenicity of a two-dose heterologous Ad26.ZEBOV and MVA-BN-Filo Ebola vaccine regimen in adults in Europe (EBOVAC2): A randomised, observer-blind, participant-blind, placebo-controlled, phase 2 trial. Lancet Infect. Dis..

[78] Tapia M.D., O Sow S., Ndiaye B.P., Mbaye K.D., Thiongane A., Ndour C.T., Mboup S., A Ake J., Keshinro B., A Akintunde G. (2020). Safety, reactogenicity, and immunogenicity of a chimpanzee adenovirus vectored Ebola vaccine in adults in Africa: A randomised, observer-blind, placebo-controlled, phase 2 trial. Lancet Infect. Dis..

[79] Sadoff J., Gray G., Vandebosch A., Cárdenas V., Shukarev G., Grinsztejn B., Goepfert P.A., Truyers C., Fennema H., Spiessens B. (2021). Safety and Efficacy of Single-Dose Ad26.COV2.S Vaccine against COVID-19. N. Engl. J. Med..

[80] Voysey M., Costa Clemens S.A., Madhi S.A., Weckx L.Y., Folegatti P.M., Aley P.K., Angus B., Baillie V.L., Barnabas S.L., Bhorat Q.E. (2021). Single-dose administration and the influence of the timing of the booster dose on immunogenicity and efficacy of ChAdOx1 nCoV-19 (AZD1222) vaccine: A pooled analysis of four randomised trials. Lancet.

[81] Folegatti P.M., Ewer K.J., Aley P.K., Angus B., Becker S., Belij-Rammerstorfer S., Bellamy D., Bibi S., Bittaye M., Clutterbuck E.A. (2020). Safety and immunogenicity of the ChAdOx1 nCoV-19 vaccine against SARS-CoV-2: A preliminary report of a phase 1/2, single-blind, randomised controlled trial. Lancet.

[82] Ewer K.J., Barrett J.R., Belij-Rammerstorfer S., Sharpe H., Makinson R., Morter R., Flaxman A., Wright D., Bellamy D., Bittaye M. (2021). T cell and antibody responses induced by a single dose of ChAdOx1 nCoV-19 (AZD1222) vaccine in a phase 1/2 clinical trial. Nat. Med..

[83] Williams K., Bastian A.R., Feldman R.A., Omoruyi E., De Paepe E., Hendriks J., Van Zeeburg H., Godeaux O., Langedijk J.P.M., Schuitemaker H. (2020). Phase 1 Safety and Immunogenicity Study of a Respiratory Syncytial Virus Vaccine with an Adenovirus 26 Vector Encoding Prefusion F (Ad26.RSV.preF) in Adults Aged >/=60 Years. J. Infect. Dis..

[84] Salisch N.C., Stephenson K.E., Williams B.K., Cox F., van der Fits L., Heerwegh D., Truyers C., Habets M.N., Kanjilal R.D.G., Larocca R.A. (2021). A Double-Blind, Randomized, Placebo-Controlled Phase 1 Study of Ad26.ZIKV.001, an Ad26-Vectored Anti-Zika Virus Vaccine. Ann. Intern. Med..

[85] Sekaly R.P. (2008). The failed HIV Merck vaccine study: A step back or a launching point for future vaccine development?. J. Exp. Med..

[86] Dicks M., Spencer A., Edwards N., Wadell G., Bojang K., Gilbert S., Hill A.V.S., Cottingham M.G. (2012). A novel chimpanzee adenovirus vector with low human seroprevalence: Improved systems for vector derivation and comparative immunogenicity. PLoS ONE.

[87] Abbink P., Lemckert A.A.C., Ewald B.A., Lynch D.M., Denholtz M., Smits S., Holterman L., Damen I., Vogels R., Thorner A.R. (2007). Comparative seroprevalence and immunogenicity of six rare serotype recombinant adenovirus vaccine vectors from subgroups B and D. J. Virol..

[88] Lawson N.D., Stillman E.A., Whitt M.A., Rose J.K. (1995). Recombinant vesicular stomatitis viruses from DNA. Proc. Natl. Acad. Sci. USA.

[89] Roberts A., Buonocore L., Price R., Forman J., Rose J.K. (1999). Attenuated vesicular stomatitis viruses as vaccine vectors. J. Virol..

[90] Martinon F., Krishnan S., Lenzen G., Magne R., Gomard E., Guillet J.G., Lévy J.P., Meulien P. (1993). Induction of virus-specific cytotoxic T lymphocytes in vivo by liposome-entrapped mRNA. Eur. J. Immunol..

[91] Rosa S.S., Prazeres D.M.F., Azevedo A.M., Marques M.P.C. (2021). mRNA vaccines manufacturing: Challenges and bottlenecks. Vaccine.

[92] Kis Z., Kontoravdi C., Shattock R., Shah N. (2021). Correction: Kis, Z.; et al. Resources, Production Scales and Time Required for Producing RNA Vaccines for the Global Pandemic Demand. Vaccines.

[93] Polack F.P., Thomas S.J., Kitchin N., Absalon J., Gurtman A., Lockhart S., Perez J.L., Pérez Marc G., Moreira E.D., Zerbini C. (2020). Safety and Efficacy of the BNT162b2 mRNA COVID-19 Vaccine. N. Engl. J. Med..

[94] Baden L.R., El Sahly H.M., Essink B., Kotloff K., Frey S., Novak R., Diemert D., Spector S.A., Rouphael N., Buddy Creech C. (2021). Efficacy and Safety of the mRNA-1273 SARS-CoV-2 Vaccine. N. Engl. J. Med..

[95] Vogel A.B., Kanevsky I., Che Y., Swanson K.A., Muik A., Vormehr M., Kranz L.M., Walzer K.C., Hein S., Güler A. (2021). BNT162b vaccines protect rhesus macaques from SARS-CoV-2. Nature.

[96] Mulligan M.J., Lyke K.E., Kitchin N., Absalon J., Gurtman A., Lockhart S., Neuzil K., Raabe V., Bailey R., Swanson K.A. (2020). Phase I/II study of COVID-19 RNA vaccine BNT162b1 in adults. Nature.

[97] Anderson E.J., Rouphael N.G., Widge A.T., Jackson L.A., Roberts P.C., Makhene M., Chappell J.D., Denison M.R., Stevens L.J., Pruijssers A.J. (2020). Safety and Immunogenicity of SARS-CoV-2 mRNA-1273 Vaccine in Older Adults. N. Engl. J. Med..

[98] Jackson L.A., Anderson E.J., Rouphael N.G., Roberts P.C., Makhene M., Coler R.N., McCullough M.P., Chappell J.D., Denison M.R., Stevens L.J. (2020). An mRNA Vaccine against SARS-CoV-2—Preliminary Report. N. Engl. J. Med..

[99] Feldman R.A., Fuhr R., Smolenov I., Ribeiro A., Panther L., Watson M., Senn J.J., Smith M., Almarsson Ö., Pujar H.S. (2019). mRNA vaccines against H10N8 and H7N9 influenza viruses of pandemic potential are immunogenic and well tolerated in healthy adults in phase 1 randomized clinical trials. Vaccine.

[100] Dolgin E. (2021). mRNA flu shots move into trials. Nat. Rev. Drug Discov..

[101] Jackson N.A.C., Kester K.E., Casimiro D., Gurunathan S., DeRosa F. (2020). The promise of mRNA vaccines: A biotech and industrial perspective. NPJ Vaccines.

[102] Pardi N., Hogan M.J., Porter F.W., Weissman D. (2018). mRNA vaccines—A new era in vaccinology. Nat. Rev. Drug Discov..

[103] Stepinski J., Waddell C., Stolarski R., Darzynkiewicz E., Rhoads R.E. (2001). Synthesis and properties of mRNAs containing the novel “anti-reverse” cap analogs 7-methyl(3′-O-methyl)GpppG and 7-methyl (3′-deoxy)GpppG. RNA.

[104] Grudzien-Nogalska E., Stepinski J., Jemielity J., Zuberek J., Stolarski R., Rhoads R.E., Darzynkiewicz E. (2007). Synthesis of anti-reverse cap analogs (ARCAs) and their applications in mRNA translation and stability. Methods Enzymol..

[105] Ramanathan A., Robb G.B., Chan S.H. (2016). mRNA capping: Biological functions and applications. Nucleic Acids Res..

[106] Furuichi Y., Miura K. (1975). A blocked structure at the 5′ terminus of mRNA from cytoplasmic polyhedrosis virus. Nature.

[107] Tanguay R.L., Gallie D.R. (1996). Translational efficiency is regulated by the length of the 3′ untranslated region. Mol. Cell Biol..

[108] Gallie D.R. (1991). The cap and poly(A) tail function synergistically to regulate mRNA translational efficiency. Genes Dev..

[109] Park J.E., Yi H., Kim Y., Chang H., Kim V.N. (2016). Regulation of Poly(A) Tail and Translation during the Somatic Cell Cycle. Mol. Cell..

[110] Presnyak V., Alhusaini N., Chen Y.-H., Martin S., Morris N., Kline N., Olson S., Weinberg D., Baker K.E., Graveley B.R. (2015). Codon optimality is a major determinant of mRNA stability. Cell.

[111] Kudla G., Murray A.W., Tollervey D., Plotkin J.B. (2009). Coding-sequence determinants of gene expression in *Escherichia coli*. Science.

[112] Kudla G., Lipinski L., Caffin F., Helwak A., Zylicz M. (2006). High guanine and cytosine content increases mRNA levels in mammalian cells. PLoS Biol..

[113] Anderson B.R., Muramatsu H., Nallagatla S.R., Bevilacqua P.C., Sansing L.H., Weissman D., Karikó K. (2010). Incorporation of pseudouridine into mRNA enhances translation by diminishing PKR activation. Nucleic Acids Res..

[114] Park J.W., Lagniton P.N.P., Liu Y., Xu R.H. (2021). mRNA vaccines for COVID-19: What, why and how. Int. J. Biol. Sci..

[115] Fan Y., Marioli M., Zhang K. (2021). Analytical characterization of liposomes and other lipid nanoparticles for drug delivery. J. Pharm. Biomed. Anal..

[116] Buschmann M.D., Carrasco M.J., Alishetty S., Paige M., Alameh M.G., Weissman D. (2021). Nanomaterial Delivery Systems for mRNA Vaccines. Vaccines.

[117] Gregory A.E., Titball R., Williamson D. (2013). Vaccine delivery using nanoparticles. Front. Cell Infect. Microbiol..

[118] Schoenmaker L., Witzigmann D., Kulkarni J.A., Verbeke R., Kersten G., Jiskoot W., Crommelin D.J.A. (2021). mRNA-lipid nanoparticle COVID-19 vaccines: Structure and stability. Int. J. Pharm..

[119] Zeng C., Zhang C., Walker P.G., Dong Y. (2020). Formulation and Delivery Technologies for mRNA Vaccines. Current Topics in Microbiology and Immunology.

[120] Kenney R.T., Frech S.A., Muenz L.R., Villar C.P., Glenn G.M. (2004). Dose sparing with intradermal injection of influenza vaccine. N. Engl. J. Med..

[121] Saroja C., Lakshmi P., Bhaskaran S. (2011). Recent trends in vaccine delivery systems: A review. Int. J. Pharm. Investig..

[122] Pardi N., Hogan M., Naradikian M.S., Parkhouse K., Cain D.W., Jones L., Moody M.A., Verkerke H.P., Myles A., Willis E. (2018). Nucleoside-modified mRNA vaccines induce potent T follicular helper and germinal center B cell responses. J. Exp. Med..

[123] O’Shea J., Prausnitz M.R., Rouphael N. (2021). Dissolvable Microneedle Patches to Enable Increased Access to Vaccines against SARS-CoV-2 and Future Pandemic Outbreaks. Vaccines.

[124] Holmgren J., Czerkinsky C. (2005). Mucosal immunity and vaccines. Nat. Med..

[125] Carter N.J., Curran M.P. (2011). Live attenuated influenza vaccine (FluMist(R); Fluenz): A review of its use in the prevention of seasonal influenza in children and adults. Drugs.

[126] Shah N.J., Najibi A.J., Shih T.-Y., Mao A.S., Sharda A., Scadden D.T., Mooney D.J. (2020). A biomaterial-based vaccine eliciting durable tumour-specific responses against acute myeloid leukaemia. Nat. Biomed. Eng..

[127] Liu H., Moynihan K., Zheng Y., Szeto G., Li A.V., Huang B., Van Egeren D., Park C., Irvine D.J. (2014). Structure-based programming of lymph-node targeting in molecular vaccines. Nature.

[128] Gustafson C.E., Kim C., Weyand C.M., Goronzy J.J. (2020). Influence of immune aging on vaccine responses. J. Allergy Clin. Immunol..

[129] Bulcha J.T., Wang Y., Ma H., Tai P.W.L., Gao G. (2021). Viral vector platforms within the gene therapy landscape. Signal. Transduct. Target. Ther..

[130] Xu S., Yang K., Li R., Zhang L. (2020). mRNA Vaccine Era-Mechanisms, Drug Platform and Clinical Prospection. Int. J. Mol. Sci..

[131] Ura T., Yamashita A., Mizuki N., Okuda K., Shimada M. (2021). New vaccine production platforms used in developing SARS-CoV-2 vaccine candidates. Vaccine.

[132] Beissert T., Perkovic M., Vogel A., Erbar S., Walzer K.C., Hempel T., Brill S., Haefner E., Becker R., Türeci Ö. (2020). A Trans-amplifying RNA Vaccine Strategy for Induction of Potent Protective Immunity. Mol. Ther..

[133] Moon J.J., Suh H., Li A.V., Ockenhouse C.F., Yadava A., Irvine D.J. (2012). Enhancing humoral responses to a malaria antigen with nanoparticle vaccines that expand Tfh cells and promote germinal center induction. Proc. Natl. Acad. Sci. USA.

[134] Miao L., Zhang Y., Huang L. (2021). mRNA vaccine for cancer immunotherapy. Mol. Cancer.

[135] Levine B.L., Miskin J., Wonnacott K., Keir C. (2017). Global Manufacturing of CAR T Cell Therapy. Mol. Ther. Methods Clin. Dev..

